# Marine Microalgal Products with Activities against Age-Related Cardiovascular Diseases

**DOI:** 10.3390/md22050229

**Published:** 2024-05-17

**Authors:** Nova Yurika, Eleonora Montuori, Chiara Lauritano

**Affiliations:** 1 Marine Biology Research Group, Ghent University, Krijgslaan 281, B-9000 Gent, Belgium; yurika.nova@ugent.be; 2 Ecosustainable Marine Biotechnology, Stazione Zoologica Anton Dohrn, Via Acton 55, 80133 Napoli, Italy; eleonora.montuori@studenti.unime.it; 3 Department of Chemical, Biological, Pharmaceutical and Environmental Sciences, University of Messina, Viale F. Stagno d’Alcontres 31, 98166 Messina, Italy

**Keywords:** cardiovascular diseases, marine microalgae, antioxidants, age-related diseases, bioactive compounds, marine natural products

## Abstract

Heart disease is one of the leading causes of death worldwide, and it is estimated that 17.9 million people die of it each year. The risk factors for cardiovascular diseases are attributable to an unhealthy and sedentary lifestyle, poor nutrition, stress, genetic predisposition, diabetes, obesity, and aging. Marine microalgae have been the subject of numerous studies for their potential activity against several human diseases. They produce a plethora of primary and secondary metabolites such as essential nutrients, vitamins, pigments, and omega-3 fatty acid. Many of these molecules have antioxidant properties and have been shown to play a role in the prevention of heart diseases. The aim of this review is to summarize recent studies on the discovery of marine microalgal compounds and bioactivities for cardiovascular diseases, including in vitro and in vivo studies, showing and discussing recent discoveries and trends. The most promising results were found for microalgal polysaccharides, peptides and carotenoids. In conclusion, the overall data summarized here show that microalgae-based supplementation has the potential to improve age-related cardiovascular diseases and we expect more clinical studies in the future.

## 1. Introduction

Marine microalgae have been the subject of numerous studies for their potential activities against several human diseases [1,2]. Microalgae have attracted a lot of attention in recent years owing to their biodiversity in terms of species, adapted to live in different environments, and in terms of chemical diversity, ranging from lipids and carbohydrates to complex polyketides. In addition, their use has been considered eco-sustainable and eco-friendly owing to their high growth rates and the possibility of culturing these both indoors and outdoors at an industrial scale. Various studies have also shown that these microorganisms are a promising source of beneficial nutrients for heart health. They produce a plethora of metabolites such as essential nutrients, vitamins, pigments, omega-3 fatty acid, and several antioxidant molecules which may play a role in the prevention of heart disease [3,4]. In particular, the n-3 long-chain polyunsaturated fatty acids (n-3 LC-PUFAs), such as eicosapentaenoic (EPA) and docosahexaenoic (DHA) acids, are known for their beneficial effects on the cardiovascular system [5,6] and to have protective effects against atherosclerotic, arrhythmic and thrombotic diseases [7,8]. These fatty acids have been reported to reduce cholesterol levels in the blood, lower blood pressure, and reduce inflammation [5,6]. The European Food Safety Authority (EFSA) recommends an intake of 250 mg for EPA plus DHA for adults, 100 mg DHA for infants (>6 months) and young children <24 months, and to increase the dose during pregnancy and lactation [9]. Furthermore, marine microalgae also contain other antioxidant molecules, such as pigments [10] and vitamins, like vitamin E, vitamin A and vitamin of complex B. These antioxidants have been reported to play a significant role in the prevention of cardiovascular diseases (CVD) [11,12]. For instance, it has been shown that pre-treatments with antioxidants, such as vitamins C and E, can mitigate endothelial dysfunction due to high-fat meals [13].

According to the World Health Organization (WHO), heart disease is one of the leading causes of death worldwide (https://www.who.int/health-topics/cardiovascular-diseases/#tab=tab_1 accessed on 29 January 2024), with about 17.9 million deaths globally each year. Cardiovascular diseases are the most prevalent age-related diseases [14,15]. As the pace of population aging around the world is increasing dramatically, old population presents one of the greatest challenges for the social and health care systems worldwide, especially in low-income and middle-income countries [16]. For older patients, hypertension, hyperlipidemia and diabetes are also frequent negatively influencing cardiovascular events [17]. Overall, cardiovascular disease prevention in older adults should be established based on the individuals, based on their estimated life expectancy, time to benefit, comorbidities, and preferences (e.g., more plant-based and low-fat diet, exercise when possible, quitting smoking, etc.).

**Figure 1:**
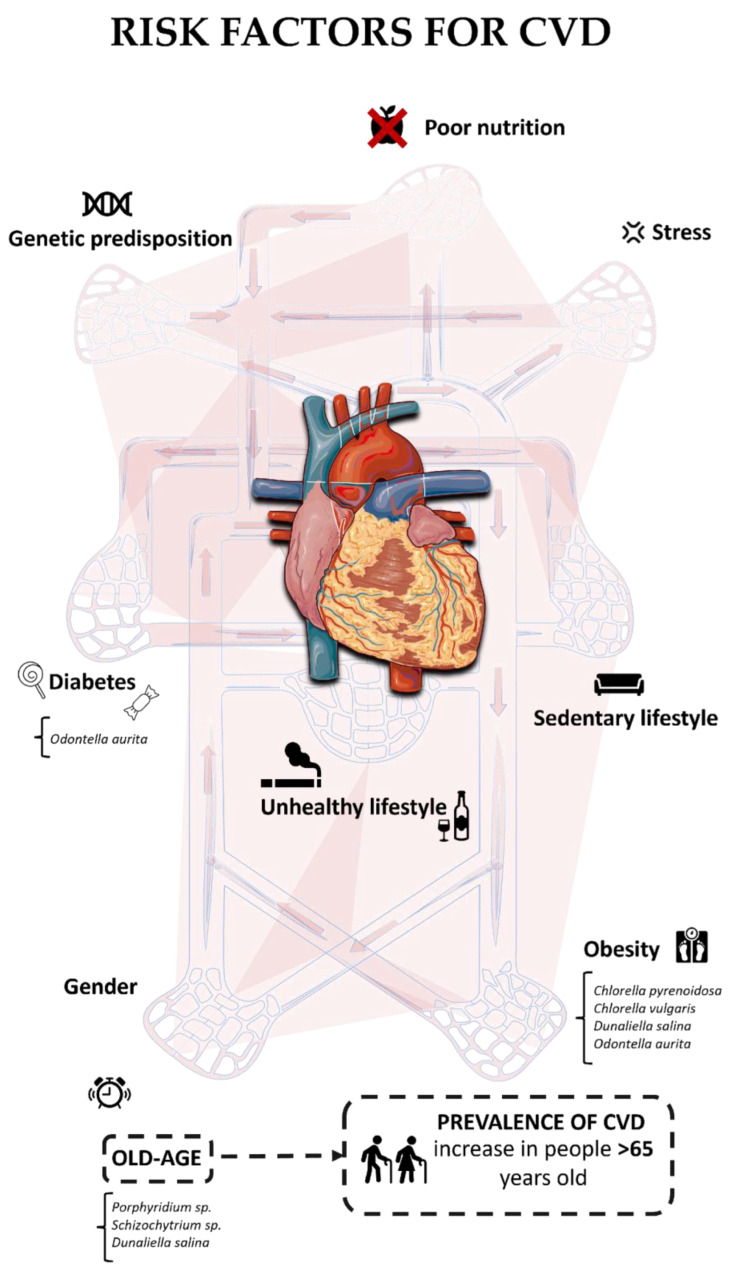
A schematic representation of principal risk factors of cardiovascular diseases and main microalgae which have shown potential beneficial activities.

The risk factors for cardiovascular diseases are mainly attributable to an unhealthy lifestyle, poor nutrition, sedentary lifestyle, stress, genetic predisposition, diabetes, obesity and aging (Figure 1) [18]. As reported in Izzo et al. [19], both age and gender are risk factors. Older females are more susceptible to cardiovascular disease compared to men of the same age. In both cases, in both men and women, these diseases are related to a decrease in sex hormones [19,20]. Possible women-specific risk factors that have been considered include gestational diabetes mellitus, pregnancy-induced hypertension, and preeclampsia, as well as reproductive endocrine disorders, including polycystic ovary syndrome and menopause [20]. CVD risk factors are highly prevalent in some countries and vary according to socioeconomic, gender, and educational levels [18]. In Pakistan, smoking (46%), family history (43%), hypertension (37%), dyslipidemia (33%), diabetes mellitus (18%) and overweight (63.3%) are the most common risk factors found in CVD patients under 45 years of age [21]. In the UK, the rate of hypertension has been reported as the highest risk, approximately 65%, followed by smoking (44.2%), high cholesterol (38.7%), diabetes (12%), overweight (5.13%), male gender (4.6%), and female gender (5.6%). In adults, metabolic risk factors tend to increase with age [22]. Additional factors, including frailty, obesity, and diabetes could complicate and enhance CVD risk factors amongst the elderly [23,24]. With advancing age, the heart undergoes structural and functional changes that make it more susceptible to pathologies such as heart failure, arrhythmia and atherosclerosis [25]. The onset of various health issues is related to the subsequent contribution of damages to the blood vessels and the heart itself. Obesity can cause an increase in blood cholesterol, which leads to a greater predisposition to the development of atherosclerotic diseases.

In this review, we reported marine microalgal compounds with beneficial and preventive activities against heart diseases related to aging. When available, we also discussed doses and mechanisms of action for both in vitro and in vivo studies. We showed that the most bioactive molecules from microalgae reported for CVDs were polysaccharides, peptides, carotenoids and lipids.

## 2. Polysaccharides

Several studies have reported the potential of polysaccharides (PSs) to improve endothelial dysfunction, defined as functional, structural, and communication changes between the vascular endothelium and muscle cells [26]. For example, Levy-Ontman et al. in 2017 [27] evaluated the anti-inflammatory and vasodilation properties of polysaccharides produced by *Porphyridium* sp. using human coronary artery endothelial cells (HCAECs). The authors showed that polysaccharides were able to attenuate inflammatory processes by interfering with tumor necrosis factor-alpha (TNF-α)-induced inflammation. In cells pre-treated with polysaccharides, there was an up-regulation of adhesion molecule 1 (ICAM-1) and vascular cell adhesion molecule 1 (VCAM-1), nuclear factor kappa-B (NF-kB) translocation, and attenuated inhibitor of nuclear factor kappa B (IκB) degradation. Polysaccharides improved endothelial function as measured by increased nitric oxide NO formation and decreased endothelin 1 (ET-1) protein expression [27]. Hamias et al. in 2018 [28] studied the ability of polysaccharides (PSs) from *Porphyridium* sp. to improve endothelial state and found that PSs attenuated inflammatory atherosclerotic pathways up-regulated by Angiotensin II (Ang II). When HCAECs were pre-treated with PSs (500 μg/mL) under Ang II induction, PSs were able to down-regulate the NF-kB activation and suppress adhesion molecule ICAM-1 and VCAM-1 up-regulation in a dose-dependent manner. Furthermore, polysaccharides enhanced nitric oxide (NO) and endothelial nitric oxide synthase (eNOS) production, and reduced ET-1 expression levels [28].

## 3. Peptides

### 3.1. In Vitro

In addition to polysaccharides, peptides can counteract the pathological processes by mimicking the function of mediators or modulating the activities and expression of mediators involved in hypertension, hypercholesterolemia, diabetes, inflammation and oxidative stress [29]. Lin et al. [30] studied *Isochrysis zhanjiangensis*, which was suggested to inhibit vascular injury and angiogenesis, and to have a protective effect on CVDs. They characterized the production and the activity of an octapeptide (ICE) isolated from *I. zhanjiangensis*, demonstrating that ICE was able to decrease ROS production in lipopolysaccharide (LPS)-induced HUVECs (concentrations of ICE 1, 10, 20, and 50 μM). The peptide could reduce cell damage by increasing the expression of antioxidant enzymes, such as the antioxidant enzymes superoxide dismutase (SOD), glutathione peroxidase-1 (GPX), and haem oxygenase 1 (HO-1). In addition, it also inhibited pro-inflammatory mediators tumor necrosis factor (TNF)-α, cytokine interleukin-6 (IL-6), and ICAM-1 [30] (Table 1). Vo et al. in 2013 [31] isolated two peptides with aminoacidic sequences of LDAVNR for peptide 1 and MMLDF for peptide 2 from the peptidic hydrolysates of *Spirulina maxima*. These peptides showed anti-inflammatory properties in histamine-induced EA.hy926 endothelial cells (used for cardiovascular disease research) with a decrease in interleukin (IL)-8 expression, measured by the ELISA assay. It is known that endothelial inflammation is a risk factor for atherosclerosis and the authors suggested these two peptides for possible anti-atherosclerotic activity [31].

Jiang et al. in 2021 [32] suggested that microalgal compounds may have great potential as a healthier anti-hypertensive treatment substitution to conventional anti-hypertensive drugs causing side effects. Hypertension, a risk factor for the development of CVDs, consists of a sustained increase in arterial pressure above 140/90 mm Hg [33]. In particular, the authors showed that peptides from microalgae are promising angiotensin-converting enzyme (ACE) inhibitors (Table 1). Renin–angiotensin–aldosterone system (RAAS) hyperactivity is involved in the progression of vascular disease. The key effector peptide of the RAAS, angiotensin II (Ang II), is generated by angiotensin I through endothelial angiotensin-converting enzyme (ACE). Inhibition of RAAS is recommended for managing most cardiovascular diseases, particularly hypertension, heart failure, acute myocardial infarction, and stroke [34]. ACE inhibitors and angiotensin receptor blockers (ARBs) are commonly prescribed medication for primary hypertension [35] and other chronic conditions, including heart failure, by reducing systolic function. Chen et al. in 2020 [36] purified and identified a peptide (PIZ protein hydrolysate) produced by *Isochrysis zhanjiangensis* that was able to inhibit ACE. The ACE activity calculated from the amount of hippuric acid liberated from hippuryl-His-Leu (HHL) showed that PIZ acts as a mixed non-competitive inhibitor of ACE at an IC_50_ value of 61.38 μM. Pretreatment with PIZ 10 μM for 24 h on human umbilical vein endothelial cells (HUVECs) inhibited the NF-κB pathway by protecting inhibitor IκBα degradation and down-regulating NF-κB expression. In addition, they showed that PIZ had modest ACE inhibitory effects due to its ability to reduce inflammatory cytokine expression (NO, COX-2, and ICAM-1) and block the production of ET-1. ICAM-1 and MCP-1 levels were significantly suppressed by PIZ in a dose-dependent manner. Cell treatment with PIZ 10 μM decreased the expression levels of inflammatory cytokines COX-2 and slightly inhibited iNOS and ET-1 production, thereby improving endothelial dysfunction, reducing oxidative stress, and decreasing the risk of hypertension [36]. Samarakoon et al. [37] showed that pepsin hydrolysate from *Nannochloropsis oculate* exhibited ACE inhibitory activity. They demonstrated that the IC_50_ values of purified ACE inhibitory peptides were 123 μM and 173 μM and identified Gly-Met-Asn-Asn-Leu-Thr-Pro (GMNNLTP; MW, 728 Da) and Leu-Glu-Gln (LEQ; MW, 369 Da) as novel peptides, respectively [37]. Wu et al. in 2015 [38] reported that a purified peptide (Tyr-Met-Gly-Leu-Asp-Leu-Lys) from *Isochrysis galbana* showed potent ACE inhibitory activity with an IC_50_ of 36.1 μM. In 2017, Heo et al. [39] conducted a study to produce an ACE inhibitory peptide from marine *Spirulina* sp. The ACE inhibitory peptide (Thr-Met-Glu-Pro-Gly-Lys-Pro) showed the strongest ACE activity at an IC_50_ value of 0.3 mg/mL. In addition, the human umbilical vein endothelial cells (HUVECs) were treated for 1 h with aliquots of purified peptide (62.5, 125 and 250 µM) and subsequently incubated for 24 h with Ang II (1 µM). They showed that ACE inhibitory peptide inhibited NO and ROS generation, and suppressed the expression of inducible nitric oxide synthase (iNOS) and ET-1 [39].

Cunha et al. in 2022 [40] also showed that water-soluble hydrolysates rich in proteins/peptides from the microalgae *Chlorella vulgaris* had anti-hypertensive potential by measuring the percentage inhibition of the ACE enzyme (IC_50_: 286 µg protein/mL) [40]. Recently, Pei et al. [41] showed that nonapeptide ETT (Glu-Met-Phe-Gly-Thr-Ser-Ser-Glu-Thr) from *Isochrysis zhanjiangensis* showed excellent effects in regulating hypertension by inhibiting ROS up-regulation of oxidized low-density lipoprotein receptor-1 (LOX-1) and ROS levels in Ang II-induced human umbilical vein endothelial cells (HUVECs). In addition, ETT inhibited the expression of various inflammatory mediators and the expression of related cytokines (IL-1β, IL-8, TNF-α, iNOS, COX-2, ET-1, AT-1) as well as cell adhesion molecules (ICAM-1 and VCAM-1) in a dose-dependent manner (10, 50, and 100 µM) [41]. Alzahrani et al. in 2018 [42] screened the anti-hypertension activities of *Nitzschia laevis* in vitro. The author showed that trypsin hydrolysates from this species had antagonist effects toward the ACE enzyme (IC_50_ 1.63 ± 0.01 mg/mL), higher than that of *Spirulina* and *Chlorella* [42]. Verspreet et al. [43] screened five microalgae (i.e., *Chlamydomonas nivalis*, *Porphyridium purpureum*, *Chlorella vulgaris*, *Nannochloropsis gaditana*, and *Scenedesmus* sp.) with respect to their ability to inhibit ACE by measuring the activity owing to an ACE-1 inhibition kit. The ACE inhibition bioassay showed that all microalgae tested inhibited ACE by 73.4–87.1% when tested at a concentration of 1 mg/mL [43].

### 3.2. In Vivo

Regarding in vivo experiments, the activities found were mainly related to anti-hypertension. Ko et al. [44] found that a purified peptide (Val–Glu–Gly–Tyr) from marine *Chlorella ellipsoidea* acted as a competitive inhibitor against ACE with an IC_50_ value of 128.4 μM. Furthermore, they tested the anti-hypertensive effects of the purified peptide by measuring the change in systolic blood pressure at 2, 4, 6 and 8 h after oral administration of the peptide (10 mg/kg of body weight) and showed that purified peptide was able to significantly decrease systolic blood pressure in rats [44].

Barkia et al. in 2019 [45] screened six strains of marine diatoms and found that papain hydrolysates had ACE inhibitory activity in vitro (2 mg/mL), with the highest activity obtained from *Bellerochea malleus*. Furthermore, in vivo assays showed that *Bellerochea malleus* hydrolysates reduced systolic and diastolic blood pressure in male spontaneously hypertensive rats after 5 days of hydrolysate treatment at doses of 75 and 100 mg/kg body weight [45]. Hayes et al. in 2023 [46] studied hydrolysate and bioactive peptides from the red microalga *Porphyridium* sp., namely, GVDYVRFF, AIPAAPAAPAGPKLY, and LIHADPPGVGL, and assessed the anti-hypertensive activity using spontaneously hypertensive rats. The *Porphyridium* sp. hydrolysate was also included in a food carrier (jelly candies; 0.5 g of the hydrolysate). Hydrolysate and hydrolysate–jelly candies reduced systolic blood pressure by −1.54 mm Hg and −6.17 mm Hg, respectively, while Captopril^®^ reduced systolic blood pressure by −18.21 mm Hg after 24 h [46].

## 4. Carotenoids

### 4.1. In Vitro

Microalgae are known to produce a variety of pigments with various color shades and biological activities, including carotenoids [3,12]. Zuluaga et al. in 2018 [47] reported astaxanthin protective actions against ischemia and reperfusion (I/R) injury. Astaxanthin also ameliorates myocardial cell oxidative stress injury [47]. Astaxanthin from the freshwater microalga *Haematococcus pluvialis* has also been shown to prevent oxidative stress on human endothelial cells (HUVECs) without toxicity up to a dose of 10 μg/mL [48].

### 4.2. In Vivo

El-baz et al. in 2018 [49] demonstrated that β-carotene rich *Dunaliella salina* carotenoid fraction (250 g/kg) as well as the whole biomass (250 mg/kg) had protective potentials against cardiac disfunction in a group of rats injected with D-galactose (200 mg/kg). *Dunaliella salina* β-carotene and biomass exhibited potent antioxidant activity and significant reducing capacity of homocysteine, IL-6 and iNOS. In another study, El-Baz et al. [50] examined the effects of zeaxanthin heneicosylate (ZH) isolated from *Dunaliella salina* on cardiac dysfunction. The study was performed in vivo in rats, by injecting D-galactose in rats for 8 weeks following orally treated with ZH (250 μg/kg) for a period of 28 days. ZH improved cardiac aging manifestation, including irregular heartbeat and increased NF-κB. ZH injected rats ameliorated NF-κB and restored superoxide dismutase (SOD). SOD is an antioxidant enzyme that has been shown to protect the heart against oxidative stress, and ischemic damage, and hypertrophy after myocardial infarction [51]. Oral administration of ZH up-regulated retinoic acid receptor alpha (RAR-α) gene expression in cardiac tissue. RAR-α plays important roles in cardiac regeneration after myocardial infarction. Depletion of RA pathway leads to cardiomyocyte apoptosis after myocardial infarction [52]. El-Baz et al. [53] conducted a study on the carotenoid rich fraction of the microalgae *Dunaliella salina* activity against inflammation-associated cardiac dysfunction in cardiac-obese rats induced by high fat diet, demonstrating that the carotenoid rich fraction increased adiponectin and glucagon serum level. The histopathological examination of rat treated with the carotenoid rich fraction showed the absence of fibrosis and severe congestion in the myocardial blood vessels [53].

## 5. Lipids and Other Bioactive Extracts and Molecules

In addition to polysaccharides, peptides and pigments, other molecules from microalgae have shown promising results. In particular, Dahli et al. [54] demonstrated that lyso-diacylglyceryltrimethylhomoserine (lyso-DGTS) isolated from *Nannochloropsis* sp. ethanolic extract might be useful for the prevention of atherosclerotic risk factors by showing increased activities of recombinant paraoxonase 1 (rePON1) lactonase [54].

A mixture of omega-3 polyunsaturated fatty acids (35%) from *Schizochytrium* sp., extra virgin olive oil (75%) and algae oil (25%) was reported to activate the phosphoinositide 3 kinase (PI3K/Akt) pathway that is known to repair vascular endothelium. Aortic rings from old rats treated with the oil mixture (2.5 mL/kg) showed a decreased response to the vasoconstrictor Ang II [55]. Haimeur et al. [56] assessed the effects of two n-3 PUFA from freeze-dried *Odontella aurita* on risk factors for CVDs. A rat group fed with the high-fat diet supplemented with *Odontella aurita* displayed a significantly lower body weight and reduced insulinemia, as well as a reduced serum lipid level, reduced platelet aggregation and oxidative status induced by high fat intake. The authors reported that *Odontella aurita* was more effective than the fish oil in reducing the hepatic triacyglycerol levels and in preventing high-fat diet-induced steatosis [56].

Dudek et al. [57] summarized, in a review, the beneficial role of dietary silicon in the prevention of age-related diseases. Vide et al. [58] reported the effects of *Spirulina* and dietary silicon-enriched *Spirulina* (SES) on atherosclerosis. Hamsters on a high-fat diet were treated with *Spirulina* or SES at a dose 57 mg/kg body weight daily, corresponding to 0.57 mg of silicon/kg body weight. The results showed that in the SES group, there was a reduction in inflammation by lowering the levels of TNF-α, IL-6, as well as a reduction in the number of polymorphonuclear cells and prevention of the activity of NF-κB. Both SES and *Spirulina* itself similarly protected against oxidative stress by reducing the activity of nicotinamide adenine dinucleotide phosphate oxidase (NOX) and maintaining the activity of the antioxidant SOD and glutathione peroxidase [58].

Quagliariello et al. in 2022 [59] reported that *Spirulina platensis*, *Ganoderma lucidum* and *Moringa oleifera* were able to improve cardiac function by reducing inflammation and cardiotoxicity induced by anthracyclines, adjuvant therapies for cancers. Female mice were treated with doxorubicin (DOXO) or a combination of *Spirulina*, *Ganoderma lucidum*, and *Moringa oleifera* (Singo). Following that, they analyzed the myocardial expressions of nucleotide-binding domain, leucine-rich–containing family, pyrin domain-containing-3 (NLRP3), galectin-3 and calgranulin S100, and 13 cytokines through ELISA methods. The authors also assessed myocardial fibrosis, necrosis, and hypertrophy through immunohistochemistry. In addition, they performed tests on human cardiomyocytes by exposing them to DOXO (200 nM) alone or in combination with Singo (at 10, 25 and 50 µg/mL) for 24 and 48 h. The results showed that Singo reduced NLRP3 and p65/NF-kB levels in human cardiomyocytes exposed to Singo at 10, 15 and 50 µg/mL and reduced cytokine levels (the concentration of Singo was 25 µg/mL). Immunohistochemistry analysis indicated that Singo (at 12 mg/kg) reduced fibrosis and hypertrophy in the myocardial tissues of mice during exposure to DOXO [59].

Umei et al. in 2022 [60] demonstrated that oral administration of *Euglena gracilis* was beneficial to improve cardiac function in a mice model of isoproterenol-induced heart failure. A group of mice were injected with isoproterenol (ISO) (20 mg/kg/day) for 7 days. They showed that oral administration of *Euglena gracilis* (2%), in combination with an AIN93G diet, alleviated cardiac dysfunction [60]. Song et al. [51] tested *Dunaliella salina*’s protective effects on myocardial ischemia/reperfusion injury (MIRI) in the Langendorff perfused heart model in mice. The authors reported that *D. salina* (500 mg/kg) was able to improve left ventricle function, reduce the rate of malignant arrhythmia and infarct size, and increase the antioxidant superoxide dismutase. In a recent study published by Tsai et al. in 2023 [61], *D. salina* was reported to have cardioprotective effects against myocardial ischemia/reperfusion (I/R) injury. A group of rats was subjected to surgical procedures for inducing myocardial I/R injury. *D. salina* extract treatment (0.1 mg/kg) was able to decrease myocardial infarct size and attenuate the expressions of cyclooxygenase-2 (COX-2) and the activity of STAT1, janus kinase 2 (JAK2), inhibitor of IκB, NF-κB [61]. Yang et al. [62] showed that *Chlorella pyrenoidosa* was able to lower the blood pressure in rats fed a diet containing N ω-nitro-L-arginine methyl ester hydrochloride (L-NAME), which induced endothelial dysfunction (40 mg/kg). Rats consuming 4 and 8% *Chlorella* had significantly lower ACE activity in the aorta and reduced TNF-α concentrations in the aorta and heart. Histopathological results showed that *Chlorella* consumption reduced the injury scale of the coronary arteries, ventricles, and septum of the heart [62].

### Clinical Studies

Recently, Sandgruber et al. [63] completed a clinical trial with 80 young and healthy participants who consumed a smoothie enriched with either 15 g of *Chlorella pyrenoidosa* dry weight (d.w.) or 15 g of *Microchloropsis salina* d.w. for 14 days. They demonstrated that regular consumption of *Chlorella pyrenoidosa* ameliorated CVD factors such as total cholesterol, LDL cholesterol, the LDL–cholesterol to HDL–cholesterol ratio, and non-HDL cholesterol, possibly due to its rich vitamin D2 source. *Microchloropsis salina* improved the fatty acid distribution in plasma lipids by increasing the LC n3 PUFA content and reducing the n6/n3 PUFA ratio [63]. Clinical studies with *Chlorella* were also conducted by Shimada et al. [64] with eighty subjects with systolic blood pressure of 130–159 mmHg or diastolic blood pressure of 85–99 mmHg. The subjects took γ-Aminobutyric Acid (GABA)-rich *Chlorella* (20 mg as γ-aminobutyric acid or placebo twice daily for 12 weeks) as a dietary supplement. Systolic blood pressure decreased significantly compared with placebo, with a higher reduction in the subjects with borderline hypertension than in the subjects with high–normal blood pressure [64]. A randomized triple-blind placebo-controlled clinical trial study conducted by Ghaem et al. [65] in 2021 involved 41 patients with hypertension consuming a salad dressing containing 2 g of *Spirulina platensis* powder for two months. The results showed that the *Spirulina* dressing significantly decreased systolic blood pressure, diastolic blood pressure, serum triglyceride, total cholesterol, and low-density lipoprotein (LDL) levels in comparison to placebo controls [65]. Bioactive compounds and extracts from microalgae for CVDs are summarized in Table 1.

**Table 1:** The table reports marine microalgal bioactive compounds with potential beneficial activities for cardiovascular diseases. Microalgae, activity observed, compound, concentration (Conc.) used, and model are reported. Abbreviations: CVDs for cardiovascular diseases, DHA for Docosahexaenoic acid, EPA for Eicosapentaenoic acid, EVOO for extra virgin olive oil, IC_50_ for inhibitory concentration values, NLRP3 for NOD-, LRR- and pyrin domain-containing protein 3.

Microalgae	Activity Observed	Compound	Conc.	Model	Reference
**Polysaccharides**					
*Porphyridium* sp. (Rhodophyta/Porphyridiophyceae)	Preserve endothelial function, anti-inflammatory	Polysaccharides	50 μg/mL	In Vitro: Human coronary artery endothelial cells (HCAECs)	[27]
*Porphyridium* sp. (Rhodophyta/Porphyridiophyceae)	Preserve endothelial function, anti-atherosclerosis	Polysaccharide	500 mg/mL	In Vitro: Human coronary artery endothelial cells (HCAEC)	[28]
**Peptides**					
*Spirulina maxima* (Cyanobacteria/Cyanophyceae)	Anti-atherosclerosis	Peptic hydrolysates of *Spirulina*	200 µM	In Vitro: EA.hy926 endothelial cell	[31]
*Isochrysis zhanjiangensis* (Haptophyta/Coccolithophyceae)	Inhibit vascular injury and angiogenesis	Octapeptide (IEC; Ile-Ile-Ala-Val-Glu-Ala-Gly-Cys)	1, 10, 20, and 50 μM	In Vitro: Human umbilical vein endothelial cells (HUVECs)	[30]
*Isochrysis zhanjiangensis* (Haptophyta/Coccolithophyceae)	Anti-hypertensive, angiotensin-converting enzyme (ACE) inhibitors	Peptide (PIZ; Phe-Glu-Ile-His-Cys-Cys)	IC_50_ = 61.38 μM	In Vitro: Hippuryl-His-Leu (HHL) HHL assay	[36]
*Chlamydomonas nivalis* (Chlorophyta/Chlorophyceae), *Porphyridium purpureum* (Rhodophyta/Porphyridiophyceae), *Chlorella vulgaris* (Chlorophyta/Trebouxiophyceae), *Nannochloropsis gaditana* (Heterokontophyta/Eustigmatophyceae), and *Scenedesmus* sp. (Chlorophyta/Chlorophyceae)	Angiotensin-converting enzyme (ACE) inhibitors	-	1 mg/mL	In Vitro: Hippuryl-His-Leu (HHL) HHL assay	[43]
*Chlorella vulgaris* (Chlorophyta/Trebouxiophyceae)	Anti-hypertensive, angiotensin-converting enzyme (ACE) inhibitors	Water-soluble hydrolysates rich in proteins/peptides	IC_50_: 286 µg protein/mL	In Vitro: Hippuryl-His-Leu (HHL) HHL assay	[40]
*Nannochloropsis oculate* (Heterokontophyta/Eustigmatophyceae)	Angiotensin-converting enzyme (ACE) inhibitors	Peptides: Gly-Met-Asn-Asn-Leu-Thr-Pro (GMNNLTP; MW, 728 Da) and Leu-Glu-Gln (LEQ; MW, 369 Da),	IC_50_: 123 IC_50_ = 173 μM, respectively	In Vitro: Hippuryl-His-Leu (HHL) HHL assay	[37]
*Nitzschia laevis* (Heterokontophyta/Bacillariophyceae)	Angiotensin-converting enzyme (ACE) inhibitors	-	IC_50_ = 1.63 ± 0.01 mg/mL	In Vitro: Hippuryl-His-Leu (HHL) HHL assay	[42]
*Isochrysis galbana* (Haptophyta/Coccolithophyceae)	Angiotensin-converting enzyme (ACE) inhibitors	Peptide: (Tyr-Met-Gly-Leu-Asp-Leu-Lys)	IC_50_ = 36.1 μM	In Vitro: Hippuryl-His-Leu (HHL) HHL assay	[38]
Marine *Spirulina* sp. (Cyanobacteria/Cyanophyceae)	Anti-hypertensive, angiotensin-converting enzyme (ACE) inhibitors	Peptide (Thr-Met-Glu-Pro-Gly-Lys-Pro)	IC_50_ = 0.3 mg/mL	In Vitro: Hippuryl-His-Leu (HHL) HHL assay	[39]
*Isochrysis zhanjiangensis* (Haptophyta/Coccolithophyceae)	Anti-atherosclerosis, anti-apoptosis and anti-inflammation	Nonapeptide named ETT (Glu-Met-Phe-Gly-Thr-Ser-SerGlu-Thr)	IC_50_ = 15.08 μM	In Vitro: Hippuryl-His-Leu (HHL) HHL assay	[41]
*Chlorella ellipsoidea* (Chlorophyta/Trebouxiophyceae)	Anti-hypertensive, angiotensin-converting enzyme (ACE) inhibitors	Peptide (Val–Glu–Gly–Tyr)	In Vitro: IC_50_ = 128.4 μM In Vivo: 10 mg/kg of body weight	In Vitro: Hippuryl-His-Leu (HHL) HHL assay In Vivo: Rats	[44]
*Bellerochea malleus* (Heterokontophyta/Mediophyceae)	Anti-hypertensive, ACE-inhibitory activities,	Papain hydrolysates	In Vitro: 2 mg m/L; In Vivo: the dose of 400 mg/kg body weight	In Vitro: Hippuryl-His-Leu (HHL) HHL assay In Vivo: Rats	[45]
*Porphyridium* sp. (Rhodophyta/Porphyridiophyceae)	Anti-hypertensive	Peptide: GVDYVRFF, AIPAAPAAPAGPKLY, and LIHADPPGVGL	-	In Vivo: Rats	[46]
**Carotenoids**					
*Dunaliella* *salina* (Chlorophyta/Chlorophyceae)	Ameliorate age-associated cardiac dysfunction	Zeaxanthin heneicosylate (ZH)	250 μg/kg	In Vivo: Rats	[49]
*Dunaliella* *salina* (Chlorophyta/Chlorophyceae)	Improve cardiac tissue fibrosis and congestion in the myocardial blood vessels	Carotenoid rich fraction	150 mg/kg body weight	In Vivo: Rats	[50]
*Haematococcus pluvialis*	Antioxidant	Astaxanthin	10 μg/mL	In Vitro: Human endothelial cells (HUVECs)	[48]
*Dunaliella salina*	Protective potentials against cardiac dysfunction Antioxidant	β-carotene rich *Dunaliella salina* carotenoid fraction	250 mg/kg	In Vivo: Rats	[53]
*Dunaliella salina* (Chlorophyta/Chlorophyceae)	Improve Myocardial ischemia-reperfusion injury (MIRI), improve left ventricle function and reduce the rate of malignant arrhythmia	-	500 mg/kg	Langendorff perfused heart model in mice	[51]
*Chlorella* sp. (Chlorophyta/Trebouxiophyceae)	Anti-hypertensive	-	20 mg	Clinical trials	[64]
*Spirulina platensis* (Cyanobacteria/Cyanophyceae)	Anti-hypertensive	-	2 g	Clinical trials	[65]
**Lipids and other bioactive extracts and molecules**					
*Nannochloropsis* sp. (Heterokontophyta/Eustigmatophyceae)	Anti-atherosclerosis	Lyso-diacylglyceryltrimethylhomoserine (lyso-DGTS)	1.43 mg/mL	In Vivo: Mice	[54]
A Mixture of *Schizochytrium* sp. and Extra Virgin Olive Oils (not found in algaebase, but found in wikipedia)	Attenuate aging-induced endothelial dysfunction	2.5 mL/kg of a mixture of 75% of EVOO (*Cornicabra* variety; 80% oleic acid and 63.49 mg/g of secoiridoids) and 25% of Algae oil (*Schizochytrium* spp.: 35% DHA, 20% EPA and 5% Docosapentaenoic (DPA))	Omega-3 polyunsaturated fatty acids (ω-3 PUFA)	In Vivo: Male Wistar rats	[55]
Freeze-dried *Odontella* *aurita* (Heterokontophyta/Mediophyceae)	Anti-atherosclerosis, reduced insulinemia, serum lipid levels, platelet aggregation and oxidative status	Marine omega-3	12% (*w*/*w*) of freeze-dried *O. aurita*	In Vivo: Male Wistar rats	[56]
*Spirulina* sp. Cyanobacteria/Cyanophyceae)	Anti-atherosclerosis	Dietary silicon-enriched *Spirulina* (SES)	Hamster on a high-fat diet were treated with *Spirulina* or SES at a dose 57 mg/kg body weight daily,	In Vivo: Hamster	[57,58]
*Spirulina platensis*, *Ganoderma lucidum* and *Moringa oleifera*	Reduction in NLRP3 and p65/NF-kB levels in human cardiomyocytes. Reduction in fibrosis and hypertrophy in the myocardial tissues of mice	Singo (*Spirulina platensis*, *Ganoderma lucidum* and *Moringa oleifera*)	In Vitro: 10, 15 and 50 µg/mL In Vivo: 12 mg/kg	In Vitro: Human cardiomyocyte. In Vivo: Mice	[59]
*Dunaliella salina*	Cardioprotective effects against myocardial ischemia/reperfusion (I/R) injury	*Dunaliella salina* extract	0.1 mg/kg	In Vivo: Rats	[61]
*Euglena gracilis*	Improvement in cardiac function	-	*Euglena gracilis* 2%	In Vivo: Mice	[66]
*Chlorella pyrenoidosa*	Ameliorative effects on CVDs factors	-	15 g for 14 days	Clinical trials	[63]
*Microchloropsis salina*	Improvement in fatty acid distribution in plasma lipids	-	15 g for 14 days	Clinical trials	[63]
*Chlorella pyrenoidosa*	Anti-hypertensive	-	40 mg/Kg	In Vivo: Rats	[62]

## 6. Conclusions

**Figure 2:**
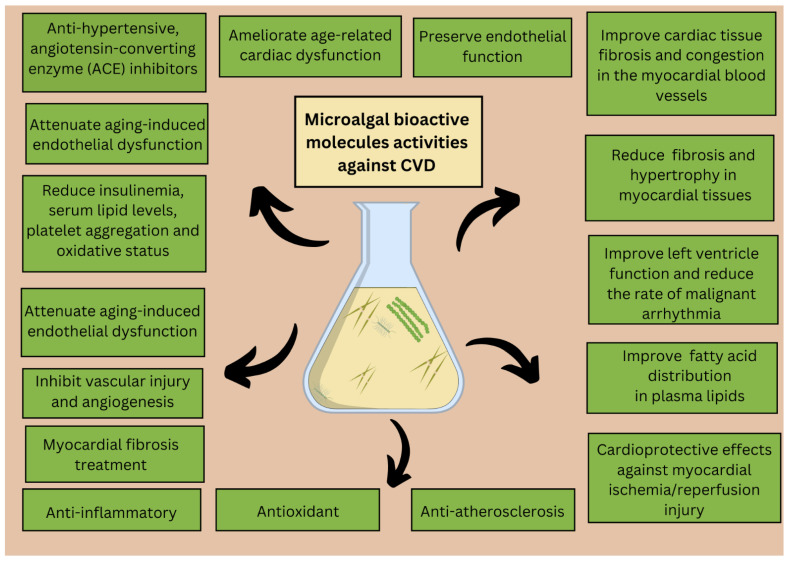
A schematic representation of microalgal bioactive molecules for different age-related cardiovascular disease applications. CVD abbreviation stands for cardiovascular disease.

Overall, this review highlights that the most common compounds with bioactivities useful for cardiovascular diseases are omega-3, pigments, peptides, and carbohydrates. The most abundant phyla of microalgae that have shown beneficial activities for heart-related diseases were Chlorophyta (i.e., *Chlorella* sp., *Chlamydomonas nivalis*, *Chlorella vulgaris*, *Chlorella ellipsoidea*, *Scenedesmus* sp., *Dunaliella salina*), followed by Heterokontophyta and Rhodophyta. In general, the most common mechanisms of action involved in the protective role of microalgal extracts and compounds for cardiovascular diseases are antioxidant and anti-inflammatory activity by reducing free radicals and inhibiting the release of inflammatory mediators (Figure 2).

As regards patents, the WO2019026067A1 relates to extracts of the microalga *Nannochloropsis* and their uses. According to the patent, the nutraceutical composition of WO2019026067A1 (https://patents.google.com/patent/WO2019026067A1/en; accessed on 14 March 2024) may be used for ameliorating conditions associated with atherogenesis and preventing atherosclerotic cardiovascular diseases and associated conditions, such as heart attack, stroke, and high blood pressure. An example of a product is Spirulysat^®^, a product produced by AlgoSource (https://algosource.com/healthcare/preventive-cardiovascular-care/; accessed on 14 March 2024), based on *Spirulina* extracts, rich in phycocyanins. AlgoSource suggests this product for cardiovascular disease prevention. In particular, Spirulysat^®^ was suggested to prevent the formation of atheroma plaques (https://algosource.com/healthcare/preventive-cardiovascular-care/; accessed on 14 March 2024). Owing to their rapid growth, the possibility of applying metabolic engineering, and multiple bioactive metabolites, marine microalgae represent a great sustainable source of molecules for an industry-scale production of ingredients for functional foods, cosmeceuticals and possible future drugs.
